# Real‐world use of maintenance bacillus Calmette‐Guérin therapy in patients with non‐muscle‐invasive bladder cancer in Japan: ASUKA study

**DOI:** 10.1002/bco2.70091

**Published:** 2025-10-01

**Authors:** Makito Miyake, Jumpei Tokumaru, Hiroshi Oi, Hiroshi Kitagawa, Kiyohide Fujimoto, Naotaka Nishiyama, Hiroshi Kitamura

**Affiliations:** ^1^ Department of Urology Nara Medical University Nara Japan; ^2^ Oncology Medical, AstraZeneca K.K. Osaka Japan; ^3^ Department of Urology, Faculty of Medicine University of Toyama Toyama Japan

**Keywords:** Bacillus Calmette Guérin vaccine, duration of treatment, Japan, non‐muscle‐invasive bladder neoplasms, observational study

## Abstract

**Objectives:**

To investigate the real‐world clinical use of maintenance bacillus Calmette‐Guérin (mBCG) therapy for high‐risk non‐muscle‐invasive bladder cancer (HR‐NMIBC) in Japan.

**Patients and methods:**

This multicentre, retrospective, observational study included patients who received intravesical mBCG for HR‐NMIBC following transurethral resection of bladder tumours between 2000 and 2023, and who were included in the Japan Urological Oncology Group registry database. Assessments included real‐world mBCG treatment duration, the completion rate of planned treatment, reasons for treatment discontinuation and mBCG effectiveness.

**Results:**

The study included 886 patients (median [interquartile range] age 71.0 [65.0–77.0] years; male, 83.5%). The median (interquartile range) treatment duration was 11 (6–17) months, with 43.8% of patients completing the physician‐determined planned treatment duration. The percentage of patients with mBCG treatment duration of 3, 6, 12, 18 and 24 months was 21.6%, 21.8%, 31.3%, 7.6% and 9.2%, respectively. Adverse events were the most common reason for mBCG discontinuation (49.1%). The recurrence‐free survival, progression‐free survival, overall survival and bladder preservation durations were numerically shorter in patients treated for 3 months.

**Conclusions:**

This first comprehensive study of the real‐world use of mBCG treatment for HR‐NMIBC in Japan found diverse treatment patterns, with approximately 40% of patients receiving mBCG for <1 year, which is shorter than the guideline‐recommended treatment duration. The results underscore the need for early and sustained adverse event management, and provide valuable reference data for optimising mBCG therapy in clinical practice.

## 1 INTRODUCTION

Approximately 75% of bladder cancer cases are non‐muscle‐invasive bladder cancer (NMIBC),^1^ among which around 50% are considered high‐risk NMIBC (HR‐NMIBC). The risk of recurrence and progression is high; approximately 60%–80% of patients experience intravesical recurrence within five years, and these recurrences can progress to muscle‐invasive bladder cancer requiring radical cystectomy.^2^ According to several guidelines, including those from the Japanese Urological Association (JUA), the standard treatment for HR‐NMIBC is transurethral resection of the bladder tumour (TURBT) followed by intravesical bacillus Calmette‐Guérin (BCG) treatment.^3^, ^4^, ^5^, ^6^ Additionally, guidelines recommend induction BCG (iBCG) treatment comprising 6–8 doses of BCG administered weekly, followed by 1–3 years of maintenance BCG (mBCG) administration. However, the lack of a strict guideline‐recommended schedule for mBCG has led to wide variability in treatment durations.^7^

Several ongoing clinical trials are evaluating immune checkpoint inhibitors in combination with mBCG to treat BCG‐naïve HR‐NMIBC,^8^, ^9^, ^10^, ^11^ and some have demonstrated significant efficacy.^12^, ^13^ An accurate understanding of real‐world mBCG treatment patterns is critical to the successful introduction of such new therapies into clinical practice, when approved, because patterns may differ from those in clinical trials. Both clinical trial and real‐world data suggest that a high incidence of adverse events (AEs) results in shorter mBCG therapy durations and poorer clinical outcomes.^14^, ^15^, ^16^ Several randomised controlled trials in Japan have reported mixed efficacy for mBCG; however, those studies are limited by the small number of patients included and lack of generalisability to real‐world populations.^17^, ^18^, ^19^ Additionally, no large‐scale, nationwide, comprehensive real‐world studies of mBCG therapy in patients with HR‐NMIBC have been conducted. In recent years, countries other than Japan have seen a BCG shortage,^20^, ^21^ resulting in a paucity of data on longer‐term mBCG therapy. For example, in a U.S.‐based insurance claims database study including data from January 2010 through February 2021, only 0.3% of patients with HR‐NMIBC completed two years of mBCG and none completed three years of mBCG therapy.^22^

To address this gap, this retrospective, observational study investigated the current real‐world clinical use of mBCG therapy for HR‐NMIBC using data from the Japan Urological Oncology Group (JUOG) registry database, the largest such registry in Japan.

## 2 PATIENTS AND METHODS

### 2.1 Study design

This was a multicentre, retrospective, observational study of patients who received intravesical mBCG for pathologically diagnosed HR‐NMIBC. Patient data were collected from the JUOG registry database,^23^ which contains data from electronic medical records at major, high‐volume urologic oncology treatment centres. A total of 31 collaborative hospitals throughout Japan contributed to the database for the entire data collection period. The database includes data for approximately 3200 patients who received intravesical BCG treatment between 2000 and 2023. Patient data before the initiation of BCG treatment (e.g., patient background, TURBT, history of NMIBC and tumour pathology), details of iBCG and/or mBCG treatments, prognosis after BCG treatment, post‐treatment after BCG failure and treatment outcome were included.

The index identification period was from the start of 2000 to the end of 2020, and the index date was defined as the date of the first iBCG administration during this period. The end of follow‐up was defined as the last confirmation date or the end of the study period, whichever came first.

### 2.2 Patients

Patients with a diagnosis of NMIBC aged ≥18 years at the date of TURBT were eligible if they were identified as high risk by the JUA Clinical Practice Guidelines for Bladder Cancer 2019 (defined as at least one of: T1, high‐grade, carcinoma in situ),^3^ were BCG‐naïve at the start of BCG treatment, and were treated with iBCG and had received at least one cycle of mBCG (defined as at least one of three doses in the first mBCG round at 3 months). Patients were excluded if they had evidence of muscle‐invasive, locally advanced, metastatic and/or extravesical bladder cancer before the index date.

This study was conducted in accordance with ethical principles outlined in the Declaration of Helsinki. The study protocol was approved by the Ethics Committee of Nara Medical University (reference protocol ID, 3781; approval date, June 3, 2024). Owing to the retrospective study design, informed consent was not obtained. Patients were informed of the opportunity to opt‐out of the database through posters and websites.

### 2.3 Outcomes

The primary outcome was the duration of real‐world mBCG treatment following TURBT. The secondary outcomes included the numbers of patients in each mBCG treatment duration category following TURBT (3, 6, 12, 18, 24, 30 or 36 months), the planned duration of mBCG treatment (determined by the physician at the start of treatment; 1, 2 or 3 years), the association between actual treatment duration and planned treatment duration, reasons for discontinuation of mBCG by treatment duration, the completion rate of planned mBCG treatment and trends in mBCG treatment duration by index year (2000–2005, 2006–2010, 2011–2015 and 2016–2020). The exploratory outcome was the effectiveness of mBCG according to treatment duration, in terms of intravesical recurrence‐free survival (RFS), high‐grade RFS, progression‐free survival (PFS; defined as progression to muscle‐invasive bladder cancer or lymph node/distant metastasis), overall survival (OS), cancer‐specific survival (CSS) and bladder preservation duration.

### 2.4 Statistical analysis

The data were analysed using descriptive summaries with the application of non‐confirmative statistical analyses; therefore, a power calculation was not required to determine sample size. Based on the feasibility assessment, the JUOG database was expected to include data from over 3000 patients with NMIBC. In Japan, 17% of patients with NMIBC who are treated with iBCG also receive mBCG.^15^ Thus, assuming that 17% of the 3000 patients in the JUOG database received mBCG, 510 mBCG‐treated patients would be available for inclusion in this study. This was considered an adequate number of patients to achieve the objective of describing the current clinical practice of mBCG for HR‐NMIBC in Japan.

Continuous variables were analysed using summary statistics, and categorical variables as frequencies and percentages. The treatment duration was grouped in categories that represent ranges of time as follows: 3 months = 3 to <6 months; 6 months = 6 to <12 months; 12 months = 12 to <18 months; 18 months = 18 to <24 months; 24 months = 24 to <30 months; 30 months = 30 to <36 months, and 36 months = ≥36 months. Time to event analysis was conducted using the Kaplan–Meier method to evaluate the treatment duration and to evaluate the treatment effectiveness, in which patients were censored at the end of follow‐up. In principle, missing data were not imputed. Incomplete dates were treated as missing except for the date of death when evaluating survival outcomes. In such cases, the imputed date of death was derived by using one for the missing day and/or January for the month. The time to event was then calculated as the death event date = max (imputed date of death, last date known to be alive). All statistical analyses were performed using GraphPad Prism version 10 (GraphPad Software, San Diego, CA, USA).

## 3 RESULTS

### 3.1 Patients

**FIGURE 1:**
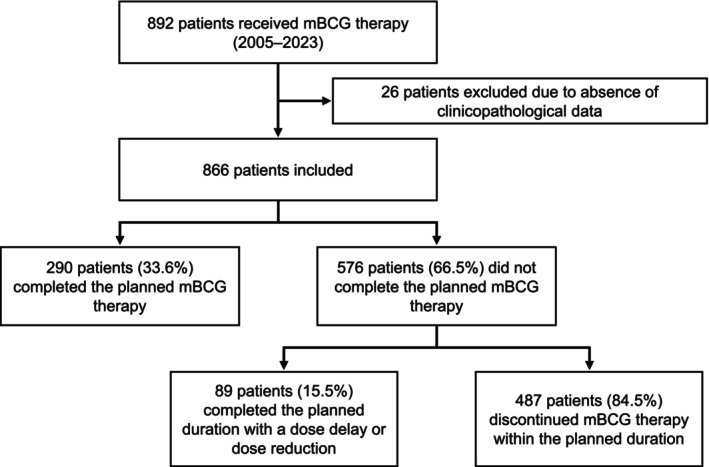
Patient disposition. Abbreviation: mBCG, maintenance bacillus Calmette‐Guérin.

A total of 866 patients were included in the study, of whom 83.5% were male; the median (interquartile range) age was 71.0 (65.0–77.0) years, and 16.7% were aged ≥80 years (Table 1). Of these patients, 83.0% received the Tokyo BCG strain, 87.1% received six iBCG treatments and 31.0% had a dose reduction of mBCG. A total of 290 patients (33.6%) completed their mBCG therapy for the duration and dosage planned by their physicians and 576 patients (66.5%) did not (including 89 patients [15.5%] with a dose reduction) (Figure 1).

**TABLE 1:** Demographics and clinical characteristics.

	Total population
	N = 866
Age, years	
Median (interquartile range)	71.0 (65.0–77.0)
Category, n (%)	
< 70 years	373 (43.1)
70 to <80 years	348 (40.2)
≥ 80 years	145 (16.7)
Sex, n (%)	
Male	723 (83.5)
Female	143 (16.5)
Smoking history, n (%)	
Never smoker	269 (31.1)
Previous smoker	430 (49.7)
Current smoker	100 (11.5)
Unknown	67 (7.7)
Body mass index, n (%)	
<18.5 kg/m^2^	68 (7.9)
18.5 to <25 kg/m^2^	574 (66.3)
≥ 25 kg/m^2^	224 (25.9)
ECOG Performance Status Scale score, n (%)	
0	824 (95.2)
1	39 (4.5)
2	2 (0.2)
3	1 (0.1)
History of NMIBC, n (%)	
Primary	670 (77.4)
Recurrent	196 (22.6)
Multiplicity, n (%)	
Single	270 (31.2)
Multiple	323 (37.3)
Unknown	273 (31.5)
Tumour size, n (%)	
< 3 cm	554 (64.0)
≥ 3 cm	159 (18.4)
Unknown	153 (17.7)
T category, n (%)	
Ta	350 (40.4)
Tis	155 (17.9)
T1	361 (41.7)
Tumour grade (WHO 2004), n (%)	
Low grade	133 (15.4)
High grade	733 (84.6)
Concurrent CIS, n (%)	
No	579 (66.9)
Yes	287 (33.1)
Variant histology, n (%)	
None	835 (96.4)
Glandular epithelium differentiation	15 (1.7)
Squamous epithelium differentiation	6 (0.7)
Micropapillary	3 (0.3)
Nested	2 (0.2)
Others	5 (0.6)
Lymphovascular invasion, n (%)	
No	856 (98.8)
Yes	10 (1.2)
Second transurethral resection, n (%)	
No	582 (67.2)
Yes	284 (32.8)
BCG strain, n (%)	
Tokyo	719 (83.0)
Connaught	147 (17.0)
Dose reduction in iBCG, n (%)	
No	680 (78.5)
Yes	186 (21.5)
Planned number of iBCG treatments, n (%)	
6	772 (89.1)
8	94 (10.9)
Actual number of iBCG treatments, n (%)	
2	1 (0.1)
4	3 (0.3)
5	18 (2.1)
6	754 (87.1)
7	9 (1.0)
8	81 (9.4)
mBCG dose reduction, n (%)	
No	597 (69.0)
Yes	269 (31.0)
Treatment following BCG failure, n (%)	
None	699 (80.7)
BCG	60 (6.9)
Chemotherapy	16 (1.8)
Radical cystectomy	26 (3.0)
Others	65 (7.5)

Abbreviations: BCG, bacillus Calmette‐Guérin; CIS, carcinoma in situ; ECOG, Eastern Cooperative Oncology Group; iBCG, induction BCG; mBCG, maintenance BCG; NMIBC, non‐muscle‐invasive bladder cancer; WHO, World Health Organization.

### 3.2 mBCG treatment duration

**FIGURE 2:**
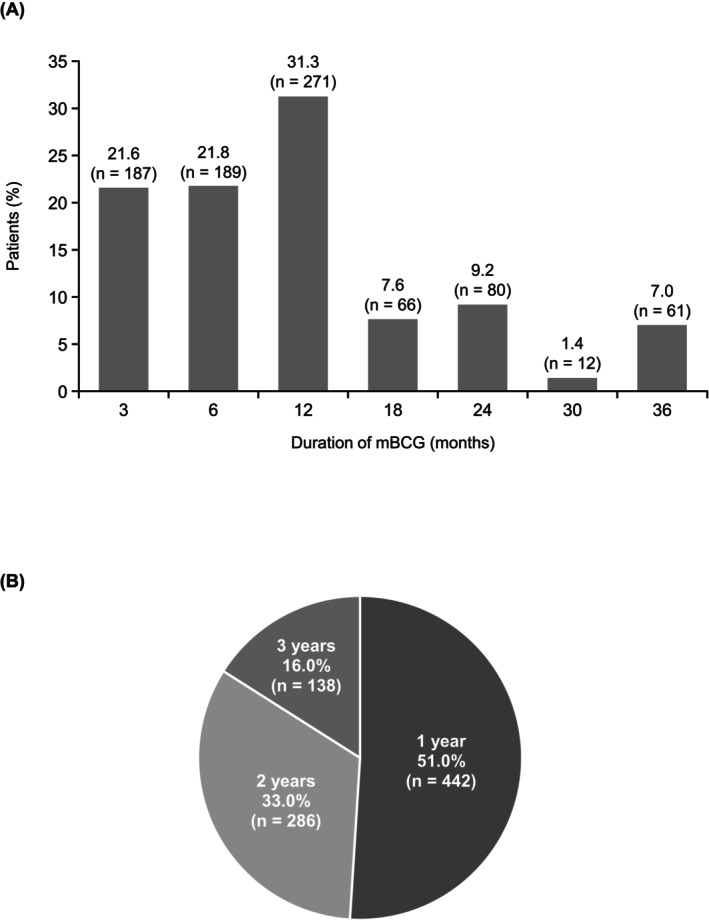
Treatment duration of mBCG therapy following transurethral resection of bladder tumour. (A) Proportion of patients in each mBCG treatment duration category. (B) Proportion of patients in each planned treatment duration category. Abbreviation: mBCG, maintenance bacillus Calmette–Guérin.

**FIGURE 3:**
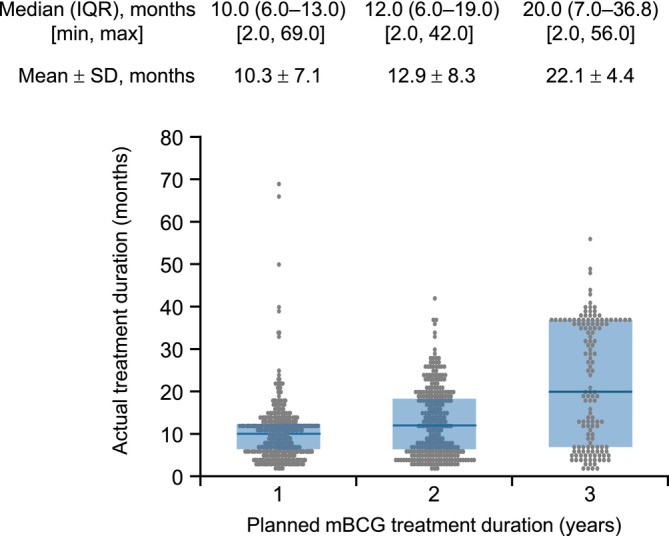
Actual mBCG treatment duration stratified by planned duration. Abbreviations: IQR, interquartile range; mBCG, maintenance bacillus Calmette–Guérin; SD, standard deviation.

The median (interquartile range) mBCG treatment duration was 11 (6–17) months. A treatment duration of ≤6 months was most common (43.4%), followed by 12 months (31.3%), 24 months (9.2%) and 18 months (7.6%) (Figure 2A). The most common planned mBCG treatment duration, as determined by the physician, was 1 year (51.0%), followed by 2 years (33.0%) and 3 years (16.0%) (Figure 2B). The median treatment duration was the longest for the group of patients with a planned treatment duration of 3 years (median [interquartile range], 20.0 [7.0–36.8] months) versus 1 (10.0 [6.0–13.0] months) or 2 years (12.0 [6.0–19.0] months) (Figure 3).

### 3.3 Completion rate

**FIGURE 4:**
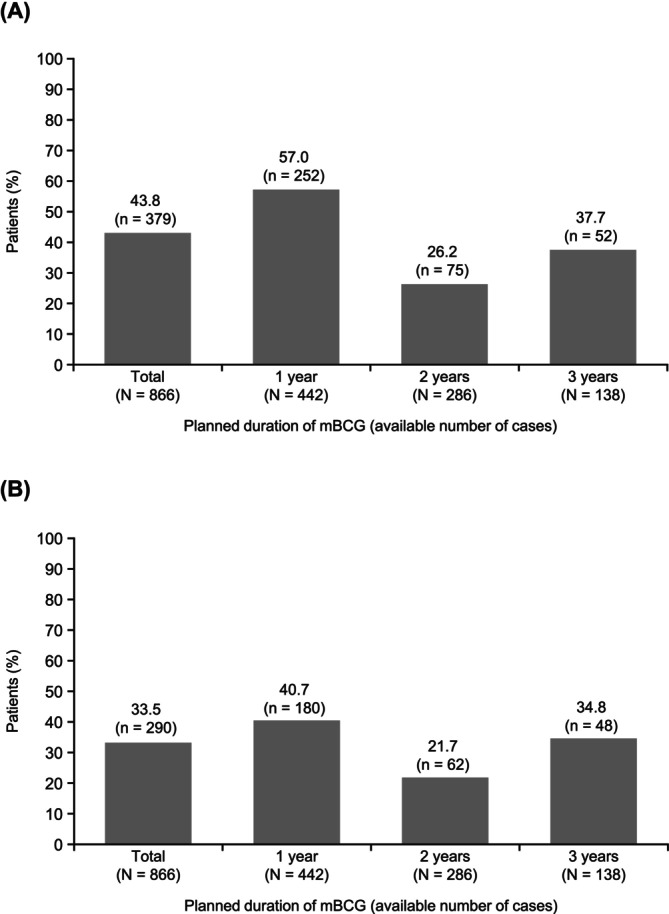
Completion rates for planned mBCG therapy duration. (A) Proportion of patients who completed mBCG therapy for the planned duration. (B) Proportion of patients who completed mBCG therapy for the planned duration without a delay or dose reduction. Abbreviation: mBCG, maintenance bacillus Calmette‐Guérin.

In the total population (N = 866), the planned duration of mBCG therapy was completed in 43.8% of patients, and 33.5% of patients completed treatment without a dose delay or dose reduction (Figure 4). Among 442 patients with a planned treatment duration of one year, the completion rate was 57.0%, and 40.7% completed treatment without a dose delay or dose reduction.

### 3.4 Reasons for mBCG discontinuation

The most common reason for mBCG discontinuation was AEs (49.1%), regardless of the duration of treatment category (Table 2). The proportion of patients who discontinued mBCG because of AEs was over 70% in the early treatment phase (3 months, 70.1%; 6 months, 74.6%). Other reasons for mBCG discontinuation were patient refusal (7.6%) and ineffective treatment (5.5%).

**TABLE 2:** Reasons for mBCG discontinuation overall and by treatment duration.

	Total	Treatment duration (months)						
		3	6	12	18	24	30	36
	N = 866	n = 187	n = 189	n = 271	n = 66	n = 80	n = 12	n = 61
Treatment ineffective	48 (5.5)	22 (11.8)	11 (5.8)	7 (2.6)	4 (6.1)	2 (2.5)	1 (8.3)	1 (1.6)
AE	425 (49.1)	131 (70.1)	141 (74.6)	85 (31.4)	39 (59.1)	16 (20.0)	7 (58.3)	6 (9.8)
Pollakiuria/urgency syndrome	202 (47.5)	61 (46.6)	70 (49.6)	42 (49.4)	20 (51.3)	6 (37.5)	2 (28.6)	1 (16.7)
Dysuria	186 (43.8)	52 (39.7)	58 (41.1)	43 (50.6)	22 (56.4)	7 (43.8)	2 (28.6)	2 (33.3)
Urinary tract infection	69 (16.2)	21 (16.0)	19 (13.5)	16 (18.8)	7 (17.9)	3 (18.8)	1 (14.3)	2 (33.3)
Fever	61 (14.4)	22 (16.8)	19 (13.5)	9 (10.6)	7 (17.9)	2 (12.5)	1 (14.3)	1 (16.7)
Macroscopic haematuria	58 (13.6)	17 (13.0)	14 (9.9)	14 (16.5)	7 (17.9)	5 (31.2)	0	1 (16.7)
Fatigue	14 (3.3)	3 (2.3)	6 (4.3)	2 (2.4)	1 (2.6)	0	1 (14.3)	1 (16.7)
Reactive arthritis	11 (2.6)	7 (5.3)	3 (2.1)	1 (1.2)	0	0	0	0
Urinary retention	7 (1.6)	1 (0.8)	4 (2.8)	0	1 (2.6)	1 (6.2)	0	0
Disseminated BCG disease	3 (0.7)	2 (1.5)	0	1 (1.2)	0	0	0	0
Refusal (could not be ruled out as related to an AE)	52 (6.0)	16 (8.6)	16 (8.5)	11 (4.1)	4 (6.1)	4 (5.0)	1 (8.3)	0
Refusal (not related to an AE)	14 (1.6)	7 (3.7)	4 (2.1)	2 (0.7)	1 (1.5)	0	0	0
Death^a^	12 (1.4)	7 (3.7)	4 (2.1)	0	1 (1.5)	0	0	0
Other	25 (2.9)	4 (2.1)	13 (6.9)	5 (1.8)	2 (3.0)	1 (1.3)	0	0

Data are n (%).

Abbreviations: AE, adverse event; BCG, bacillus Calmette‐Guérin; mBCG, maintenance BCG.

^a^ During the study period.

### 3.5 Patterns of mBCG use over time

**FIGURE 5:**
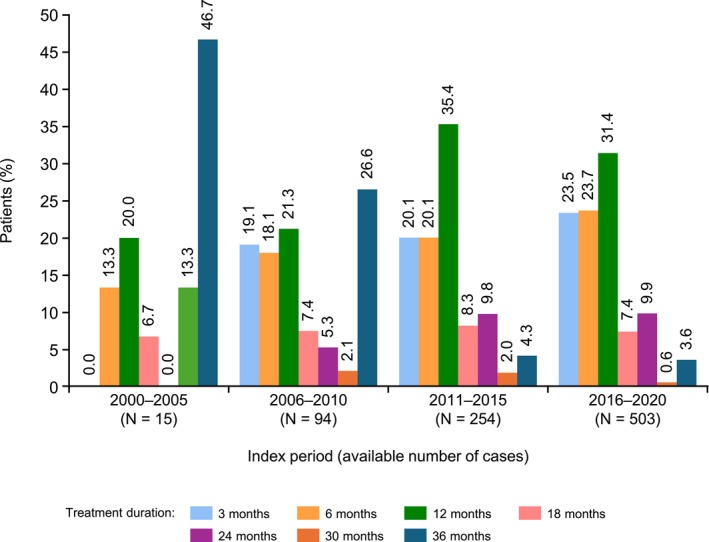
Trends in treatment duration in patients receiving real‐world mBCG therapy by index year. Abbreviation: mBCG, maintenance bacillus Calmette‐Guérin.

The number of patients who received mBCG increased over time (Figure 5). An mBCG treatment duration of 36 months was most common in the 2000–2005 (46.7%) and 2006–2010 (26.6%) periods, while a treatment duration of 12 months was most common in the 2011–2015 (35.4%) and 2016–2020 (31.4%) periods.

### 3.6 Effectiveness according to the mBCG treatment duration

**FIGURE 6:**
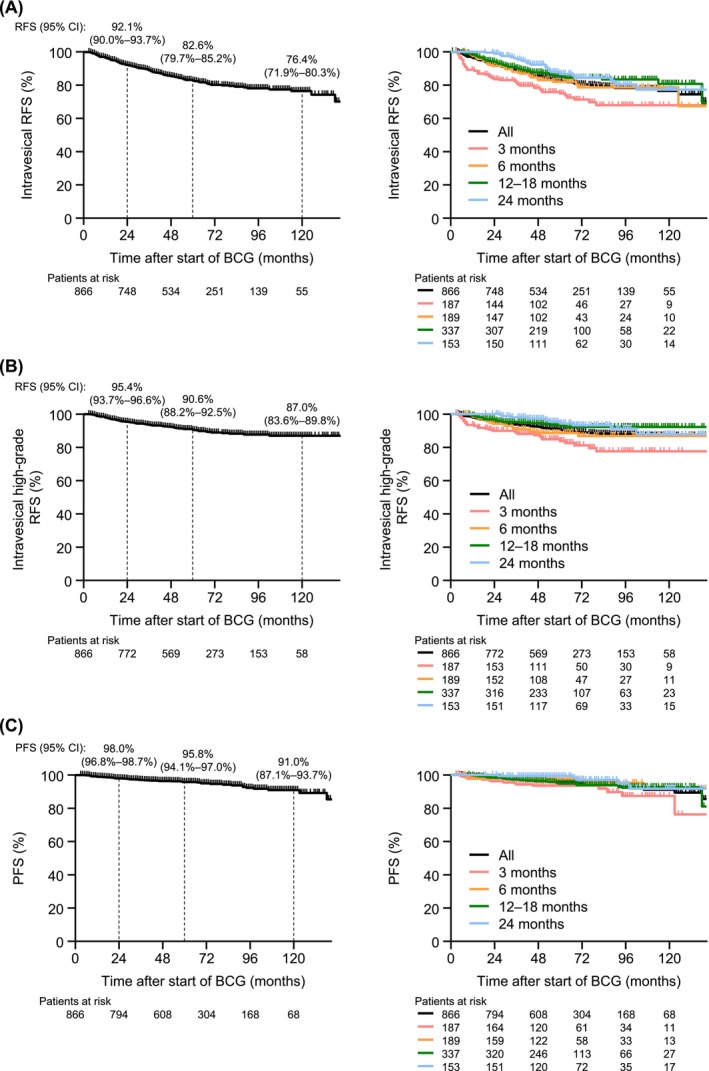
RFS and PFS of real‐world mBCG therapy. (A) Intravesical RFS. (B) Intravesical high‐grade RFS. (C) PFS (defined as progression to muscle‐invasive bladder cancer or lymph node/remote metastasis). Abbreviations: BCG, bacillus Calmette‐Guérin; CI, confidence interval; mBCG, maintenance BCG; PFS, progression‐free survival; RFS, recurrence‐free survival.

**FIGURE 7:**
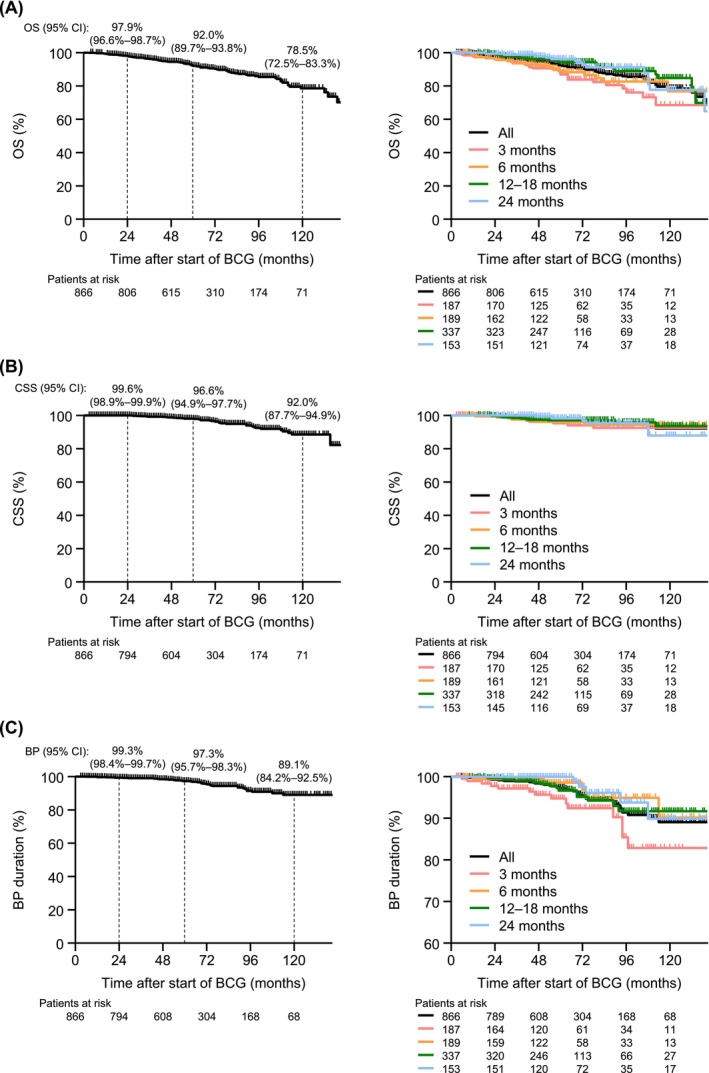
Effectiveness of real‐world mBCG therapy. (A) OS. (B) CSS. (C) BP duration. Abbreviations: BCG, bacillus Calmette‐Guérin; BP, bladder preservation; CI, confidence interval; CSS, cancer‐specific survival; mBCG, maintenance BCG; OS, overall survival.

The results of the exploratory effectiveness analysis according to the mBCG treatment duration are shown in Figures 6 and 7. Except for CSS, effectiveness was numerically shorter with a 3‐month mBCG treatment duration compared with longer treatment durations. At 24 months, all‐grade RFS was 83.4% for patients with a 3‐month treatment duration and ranged from 91.4% to 98.7% for patients with longer treatment durations. A similar trend was observed for high‐grade RFS (90.0% vs 94.2%–100%), PFS (96.1% vs 97.8%–100%), OS (96.2% vs 96.1%–99.3%) and bladder preservation (97.7% vs 99.3%–100%). This was also observed at 60 months (all‐grade RFS, 74.7% vs 81.8%–86.7%; high‐grade RFS, 83.9% vs 88.6%–94.4%; PFS, 93.5% vs 94.7%–99.3%; OS, 86.9% vs 89.3%–96.1%; bladder preservation, 94.7% vs 96.7%–100%).

## 4 DISCUSSION

This is the first study to comprehensively report the use of mBCG therapy for HR‐NMIBC in the real‐world clinical setting. Previously, the detailed status of mBCG therapy had been largely unknown, both in Japan and globally. In the JUOG database, which included over 3000 patients with NMIBC who received intravesical BCG therapy between 2000 and 2023, only 866 patients received mBCG in addition to iBCG, indicating that mBCG was administered to a limited population despite being recommended in treatment guidelines.^3^, ^4^, ^5^, ^6^ The mBCG treatment duration varied widely among the 866 patients. Around 40% of patients discontinued mBCG in <1 year. AEs were the most common reason for mBCG discontinuation and were most frequent in the early treatment phase. The exploratory analysis results indicate that the RFS, PFS, OS and bladder preservation durations were numerically shorter in patients treated for three months.

The guideline‐recommended duration of mBCG therapy is 1–3 years both in Japan and globally.^3^, ^4^, ^5^, ^6^ However, in the present study, the duration of mBCG therapy varied widely, with a median duration of 11 months. Notably, more patients received mBCG for 12 months during the 2011–2015 and 2016–2020 periods than in earlier periods. This shift may be attributed to the updated 2009 JUA guideline recommending mBCG therapy,^24^ which prompted increased adoption of the one‐year mBCG treatment protocol^25^ over the previously established three‐year protocol.^14^, ^26^ Nevertheless, the most common treatment duration was ≤6 months (43.4%) while 31.3% of patients had a treatment duration of 12 months. Similarly, a retrospective study of patients receiving monthly mBCG at two centres in Finland reported a median duration of 13 months,^27^ and a recent multinational real‐world survey of 269 patients undergoing mBCG therapy for HR‐NMIBC reported a median duration of 336 days.^28^ These results indicate a similar trend to that observed in the present study. It is important for physicians to develop individualised treatment plans based on each patient's condition and preferences, which may influence the treatment duration. In fact, patients whose planned treatment duration was three years had the longest treatment durations in the present study, with a median duration of 20.0 months.

To sustain the guideline‐recommended duration, it is essential to understand the reasons for treatment discontinuation. This study revealed that AEs were the most common reason for treatment discontinuation. Notably, the discontinuation rate due to AEs in this real‐world cohort (49.1%) was higher than that reported in the CREST trial (iBCG plus mBCG group, 9.7%).^13^ This discrepancy likely reflects that in clinical trials, patients are closely monitored and managed according to strict protocols, which may facilitate continuation despite AEs. Conversely, in real‐world practice, physicians adopt a more individualised approach and may discontinue treatment even for relatively mild AEs based on patient preferences and overall tolerability. The identification of specific reasons for discontinuation due to AEs in real‐world settings may provide useful insights for reviewing and improving current supportive care and management strategies for patients.

In the present study, the mBCG completion rate without a dose delay or dose reduction was 33.5%, and the overall completion rate was 43.8%. Although treatment discontinuation due to AEs was frequently observed, the completion rate among patients with a planned mBCG treatment duration of three years was 37.7%; however, we note the small number of patients in this group (n = 138). This is higher than the three‐year completion rate (16%) reported in a Southwest Oncology Group study (SWOG‐8507).^14^ This may be attributed to improvements in AE management and supportive care since the SWOG‐8507 study. A previous meta‐analysis reported a similar incidence of side effects for mBCG treatment durations of one and three years.^29^ However, in the current study, AEs leading to discontinuation were overwhelmingly reported within the first six months of mBCG therapy. AEs such as pollakiuria/urgency syndrome, dysuria, urinary tract infection and fever were reported to result in treatment discontinuation. Although supportive care, such as increased liquid intake to flush the bladder, over‐the‐counter pain relievers and prescription of medications to manage the side effects of BCG treatment, remains standard practice,^30^ the current findings highlight not only the need for additional options to enhance AE management, but also the importance of developing safer treatment alternatives.

This study also revealed a good overall effectiveness for mBCG treatment. The RFS, PFS and OS rates for the total population were longer than those reported in the SWOG‐8507 study (treatment duration of 36 months).^14^ The higher completion rate observed in this study may suggest that improvements in management have contributed to this outcome. The RFS rate at 24 months was similar to that reported in a Japanese study that evaluated mBCG over an 18‐month period (92.1% vs 92.7%).^17^ However, in the present study, the RFS, PFS, OS and bladder preservation durations were numerically shorter in patients treated for three months. Considering that the pattern of AEs leading to discontinuation was similar among different treatment durations, including 3 months, management strategies addressing these AEs from the early phase of mBCG therapy should be implemented to prolong treatment duration.

Some study limitations should be considered. Given the retrospective nature of this study, the potential for selection bias exists because the treatment criteria, dosage and schedule of BCG treatment are based on a limited number of individual institutional protocols and the physician's discretion. Furthermore, changes in treatment strategies during the study period may have affected the outcomes. The JUOG registry database contains limited information related to medical history and concomitant medications. Thus, the log‐rank test could not be used because it was not possible to adequately adjust for variation in background factors. Between 2000 and 2005, some institutions did not use electronic medical records, limiting data acquisition during this period. Despite these limitations, this study provides valuable reference data for future clinical trials with an mBCG control arm.

In conclusion, this is the first study to comprehensively report the status of mBCG use following TURBT in patients with HR‐NMIBC in the real‐world setting. Our findings revealed diverse treatment patterns, with approximately 40% of patients receiving mBCG for <1 year, which is shorter than the duration recommended in current guidelines. The results also indicate that AEs leading to discontinuation often occur relatively early in the treatment phase, underscoring the need for ongoing AE management from the initiation of mBCG therapy to maintain an adequate treatment duration. Importantly, our data provide insights into when and why discontinuations occur, which can support strategies to optimise patient experience and potentially reduce unnecessary treatment interruptions. The results of this study provide important real‐world reference data on mBCG therapy, thereby contributing to a better understanding of its effectiveness and safety in clinical practice.

## AUTHOR CONTRIBUTIONS

*Study conception*: Miyake, Tokumaru, Oi, Kitagawa, Nishiyama, Kitamura. *Study design*: Miyake, Tokumaru, Oi, Kitagawa, Nishiyama, Kitamura. *Data acquisition*: Miyake, Fujimoto, Nishiyama, Kitamura. *Data analysis*: Miyake, Fujimoto. *Data interpretation*: Miyake, Tokumaru, Oi, Kitagawa, Fujimoto, Nishiyama, Kitamura. *Drafting of the manuscript*: Miyake. *Critical review of the manuscript*: Miyake, Tokumaru, Oi, Kitagawa, Fujimoto, Nishiyama, Kitamura. *Final approval of the manuscript for submission*: Miyake, Tokumaru, Oi, Kitagawa, Fujimoto, Nishiyama, Kitamura. *Accountable for the accuracy and integrity of the manuscript*: Miyake, Tokumaru, Oi, Kitagawa, Fujimoto, Nishiyama, Kitamura.

## CONFLICT OF INTEREST STATEMENT

Makito Miyake has received grants or contracts from Marukai Corporation and SBI Pharmaceuticals Co. Ltd.; consulting fees from Astellas Pharma Inc., AstraZeneca K.K., MSD K.K., Pfizer Inc., Ferring Pharmaceuticals Co. Ltd., Johnson & Johnson/Janssen Pharmaceutical K.K. and Merck Biopharma Co. Ltd.; and payment or honoraria from Astellas Pharma Inc., AstraZeneca K.K., Bristol‐Myers Squibb, Ono Pharmaceutical Co. Ltd., Takeda, Pfizer Inc., Johnson & Johnson/Janssen Pharmaceutical K.K., Nippon Kayaku, Merck Biopharma Co. Ltd. and MSD K.K. Jumpei Tokumaru, Hiroshi Oi and Hiroshi Kitagawa have received support from AstraZeneca K.K. related to this manuscript. Kiyohide Fujimoto has received grants or contracts from MSD K.K., AstraZeneca K.K., Eisai Co. Ltd., Johnson & Johnson/Janssen Pharmaceutical K.K., Kissei Pharmaceutical Co. Ltd., Bristol‐Myers Squibb, SBI Pharmaceuticals Co. Ltd., and Tsumura & Co.; and payment or honoraria from MSD K.K., Merck Biopharma Co. Ltd., Janssen Pharmaceutical K.K., Kissei Pharmaceutical Co. Ltd., Bayer Yakuhin Ltd., Nippon Kayaku, Ono Pharmaceutical Co. Ltd. and Astellas Pharma Inc. Naotaka Nishiyama has no conflicts of interest to declare. Hiroshi Kitamura has received grants or contracts from AstraZeneca K.K., Bristol‐Myers Squibb, Johnson & Johnson/Janssen Pharmaceutical K.K., MSD K.K. and Takeda; consulting fees from Astellas Pharma Inc., AstraZeneca K.K., Daiichi‐Sankyo, Eisai Co. Ltd., Ferring Pharmaceuticals Co. Ltd., Johnson & Johnson/Janssen Pharmaceutical K.K., Kissei Pharmaceutical Co. Ltd., Novartis and Takeda; and payment or honoraria from Astellas Pharma Inc., AstraZeneca K.K., Bristol‐Myers Squibb, Chugai, Eisai Co. Ltd., Ferring Pharmaceuticals Co. Ltd., Johnson & Johnson/Janssen Pharmaceutical K.K., Nippon Shinyaku, Merck Biopharma Co. Ltd., MSD K.K., Nippon Kayaku, Pfizer Inc., Sanofi K.K. and Takeda.

## Data Availability

The datasets used and/or analysed during the current study are available from the corresponding author on reasonable request.

## References

[1] Matulewicz RS , Steinberg GD . Non‐muscle‐invasive bladder cancer: Overview and contemporary treatment landscape of neoadjuvant chemoablative therapies. Rev Urol. 2020;22(2):43–51. 32760227 PMC7393683

[2] Ritch CR , Velasquez MC , Kwon D , Becerra MF , Soodana‐Prakash N , Atluri VS , et al. Use and validation of the AUA/SUO risk grouping for nonmuscle invasive bladder cancer in a contemporary cohort. J Urol. 2020;203(3):505–511. 10.1097/JU.0000000000000593 31609178

[3] Japanese Urological Association . Clinical Practice Guideline for Bladder Cancer 2019 Edition Tokyo: Igakutosho‐shuppan Ltd; 2019.

[4] Holzbeierlein J , Bixler BR , Buckley DI , Holzbeierlein JM , Chang SS , Holmes R , et al. Diagnosis and treatment of non‐muscle invasive bladder cancer: AUA/SUO guideline: 2024 amendment. J Urol. 2024;211(4):533–538. 10.1097/JU.0000000000003846 38265030

[5] Powles T , Bellmunt J , Comperat E , De Santis M , Huddart R , Loriot Y , et al. Bladder cancer: ESMO clinical practice guideline for diagnosis, treatment and follow‐up. Ann Oncol. 2022;33(3):244–258. 10.1016/j.annonc.2021.11.012 34861372

[6] European Association of Urology . Non‐muscle‐invasive bladder cancer. Accessed March 2, 2025. https://uroweb.org/guidelines/non-muscle-invasive-bladder-cancer

[7] Musat MG , Kwon CS , Masters E , Sikirica S , Pijush DB , Forsythe A . Treatment outcomes of high‐risk non‐muscle invasive bladder cancer (HR‐NMIBC) in real‐world evidence (RWE) studies: Systematic literature review (SLR). Clinicoecon Outcomes Res. 2022;14:35–48. 10.2147/CEOR.S341896 35046678 PMC8759992

[8] Kamat AM , Shore N , Hahn N , Alanee S , Nishiyama H , Shariat S , et al. KEYNOTE‐676: phase III study of BCG and pembrolizumab for persistent/recurrent high‐risk NMIBC. Future Oncol. 2020;16(10):507–516. 10.2217/fon-2019-0817 32162533

[9] Steinberg GD , Shore ND , Redorta JP , Galsky MD , Bedke J , Ku JH , et al. CREST: phase III study of sasanlimab and Bacillus Calmette‐Guérin for patients with Bacillus Calmette‐Guérin‐naïve high‐risk non‐muscle‐invasive bladder cancer. Future Oncol. 2024;20(14):891–901. 10.2217/fon-2023-0271 38189180

[10] De Santis M , Abdrashitov R , Hegele A , Kolb M , Parker S , Palou Redorta J , et al. A phase 3, randomized, open‐label, multicenter, global study of durvalumab and bacillus calmette‐guérin (BCG) versus BCG alone in high‐risk, BCG‐naïve non–muscle‐invasive bladder cancer (NMIBC) patients (POTOMAC). J Clin Oncol. 2019;37(7_suppl):TPS500. Presented at the 2019 Genitourinary Cancers Symposium. 10.1200/JCO.2019.37.7_suppl.TPS500

[11] Roupret M , Neuzillet Y , Bertaut A , Pignot G , Houede N , Champiat S , et al. ALBAN: An open‐label, randomized, phase III trial evaluating the efficacy of atezolizumab in addition to one year BCG (bacillus Calmette‐Guérin) bladder instillation in BCG‐naive patients with high‐risk non–muscle‐invasive bladder cancer (AFU‐GETUG 37). J Clin Oncol. 2019;37(15_suppl):TPS4589. Presented at the 2019 ASCO Annual Meeting.

[12] AstraZeneca . Imfinzi regimen demonstrated statistically significant and clinically meaningful improvement in disease‐free survival for high‐risk non‐muscle‐invasive bladder cancer in POTOMAC phase III trial. News release. Accessed July 11, 2025. https://tinyurl.com/y6phhzhu

[13] Shore ND , Powles TB , Bedke J , Galsky MD , Palou Redorta J , Ku JH , et al. Sasanlimab plus BCG in BCG‐naive, high‐risk non‐muscle invasive bladder cancer: the randomized phase 3 CREST trial. Nat Med. 2025;31(8):2806–2814. 10.1038/s41591-025-03738-z 40450141 PMC12353837

[14] Lamm DL , Blumenstein BA , Crissman JD , Montie JE , Gottesman JE , Lowe BA , et al. Maintenance bacillus Calmette‐Guerin immunotherapy for recurrent TA, T1 and carcinoma in situ transitional cell carcinoma of the bladder: A randomized Southwest Oncology Group Study. J Urol. 2000;163(4):1124–1129. 10.1016/S0022-5347(05)67707-5 10737480

[15] Miyake M , Iida K , Nishimura N , Miyamoto T , Fujimoto K , Tomida R , et al. Non‐maintenance intravesical Bacillus Calmette‐Guérin induction therapy with eight doses in patients with high‐ or highest‐risk non‐muscle invasive bladder cancer: A retrospective non‐randomized comparative study. BMC Cancer. 2021;21(1):266. 10.1186/s12885-021-07966-7 33706705 PMC7948348

[16] Takeda T , Kikuchi E , Yuge K , Matsumoto K , Miyajima A , Nakagawa K , et al. Discontinuance of bacille Calmette‐Guérin instillation therapy for nonmuscle‐invasive bladder cancer has negative effect on tumor recurrence. Urology. 2009;73(6):1318–1322. 10.1016/j.urology.2008.12.039 19232694

[17] Hinotsu S , Akaza H , Naito S , Ozono S , Sumiyoshi Y , Noguchi S , et al. Maintenance therapy with bacillus Calmette‐Guérin Connaught strain clearly prolongs recurrence‐free survival following transurethral resection of bladder tumour for non‐muscle‐invasive bladder cancer. BJU Int. 2011;108(2):187–195. 10.1111/j.1464-410X.2010.09891.x 21176079

[18] Koga H , Ozono S , Tsushima T , Tomita K , Horiguchi Y , Usami M , et al. Maintenance intravesical bacillus Calmette‐Guérin instillation for Ta, T1 cancer and carcinoma in situ of the bladder: Randomized controlled trial by the BCG Tokyo Strain Study Group. Int J Urol. 2010;17(9):759–766. 10.1111/j.1442-2042.2010.02584.x 20604814

[19] Nakai Y , Anai S , Tanaka N , Chihara Y , Haramoto M , Otani T , et al. Insignificant role of bacillus Calmette‐Guérin maintenance therapy after complete transurethral resection of bladder tumor for intermediate‐ and high‐risk non‐muscle‐invasive bladder cancer: Results from a randomized trial. Int J Urol. 2016;23(10):854–860. 10.1111/iju.13167 27416975

[20] Balasubramanian A , Gunjur A , Weickhardt A , Papa N , Bolton D , Lawrentschuk N , et al. Adjuvant therapies for non‐muscle‐invasive bladder cancer: Advances during BCG shortage. World J Urol. 2022;40(5):1111–1124. 10.1007/s00345-021-03908-x 35083522

[21] Perera M , Papa N , Christidis D , McGrath S , Manning T , Roberts M , et al. The impact of the global bacille Calmette‐Guérin shortage on treatment patterns: Population‐based data. BJU Int. 2018;121(2):169–172. 10.1111/bju.14065 29072817

[22] Gaylis FD , Emond B , Manceur AM , Tardif‐Samson A , Morrison L , Pilon D , et al. Adherence to first‐line intravesical bacillus Calmette‐Guérin Therapy in the context of guideline recommendations for US patients with high‐risk non‐muscle invasive bladder cancer. J Health Econ Outcomes Res. 2024;11(2):109–117. 10.36469/001c.124208 39479557 PMC11523569

[23] Iida K , Miyake M , Murakami K , Komiyama M , Okajima E , Sazuka T , et al. Bacillus Calmette‐Guérin‐unresponsive non‐muscle invasive bladder cancer outcomes in patients without radical cystectomy. Int J Clin Oncol. 2021;26(11):2104–2112. 10.1007/s10147-021-01988-8 34313904

[24] Committee for Establishment of the Clinical Practice Guidelines for the Management of Bladder Cancer and the Japanese Urological Association . Evidence‐based clinical practice guidelines for bladder cancer (Summary ‐ JUA 2009 Edition). Int J Urol. 2010;17(2):102–124. 10.1111/j.1442-2042.2010.02486.x 20377834

[25] Oddens J , Brausi M , Sylvester R , Bono A , van de Beek C , van Andel G , et al. Final results of an EORTC‐GU cancers group randomized study of maintenance bacillus Calmette‐Guérin in intermediate‐ and high‐risk Ta, T1 papillary carcinoma of the urinary bladder: one‐third dose versus full dose and 1 year versus 3 years of maintenance. Eur Urol. 2013;63(3):462–472. 10.1016/j.eururo.2012.10.039 23141049

[26] Sylvester RJ , Brausi MA , Kirkels WJ , Hoeltl W , Calais da Silva F , Powell PH , et al. EORTC Genito‐Urinary Tract Cancer Group. Long‐term efficacy results of EORTC genito‐urinary group randomized phase 3 study 30911 comparing intravesical instillations of epirubicin, bacillus Calmette‐Guérin, and bacillus Calmette‐Guérin plus isoniazid in patients with intermediate‐ and high‐risk stage Ta T1 urothelial carcinoma of the bladder. Eur Urol. 2010;57(5):766–773. 10.1016/j.eururo.2009.12.024 20034729 PMC2889174

[27] Nurminen P , Nummi A , Kesti O , Ettala O , Högerman M , Järvinen R , et al. Comparison of bacillus Calmette‐Guérin maintenance therapy with monthly instillations and the Southwest Oncology Group Protocol in the treatment of non‐muscle‐invasive bladder cancer. Eur Urol Focus. 2023;9(6):1000–1007. 10.1016/j.euf.2023.04.012 37169643

[28] Bedke J , Eccleston A , Brinkmann J , Chang J , Koshy A , Milloy N , et al. Global real‐world patients characteristics, treatment patterns, and impact of BCG shortage in patients with high‐risk non‐muscle invasive bladder cancer. J Clin Oncol. 2025;43(5_suppl):717. 10.1200/JCO.2025.43.5_suppl.717

[29] Huang Z , Liu H , Wang Y , Zhang C , Xu T . Determining optimal maintenance schedules for adjuvant intravesical bacillus Calmette‐Guerin immunotherapy in non‐muscle‐invasive bladder cancer: a systematic review and network meta‐analysis. Curr Med Res Opin. 2017;33(8):1379–1387. 10.1080/03007995.2017.1326889 28471272

[30] Cleveland Clinic . Bacillus Calmette‐Guerin (BCG) treatment. Accessed May 7, 2025. https://my.clevelandclinic.org/health/treatments/17908-bacillus-calmette-guerin-bcg-treatment

